# Editorial: Tissue microenvironment in kidney diseases

**DOI:** 10.3389/fmed.2023.1134043

**Published:** 2023-02-06

**Authors:** Yuan Gui, Silvia Liu, Haiyan Fu, Dong Zhou

**Affiliations:** ^1^Division of Nephrology, Department of Medicine, University of Connecticut School of Medicine, Farmington, CT, United States; ^2^Department of Pathology, University of Pittsburgh School of Medicine, Pittsburgh, PA, United States; ^3^State Key Laboratory of Organ Failure Research, National Clinical Research Center of Kidney Disease, Division of Nephrology, Nanfang Hospital, Southern Medical University, Guangzhou, China

**Keywords:** kidney local microenvironment, cell injury/repair, cell metabolism, microbiome, cell-cell communications, cell senescence

Kidney diseases are a series of refractory syndromes that cause inevitable and irreversible clinical consequences. It is estimated that chronic kidney disease (CKD) will be the 5th most common cause of death globally by 2040 (1). Given that kidneys have limited capacities for repair and regeneration, how to prevent or mitigate kidney diseases effectively is a tough problem that physicians and scientists have faced for a long time. Over decades, substantial research has linearly advanced our recognition of kidney disease pathogenesis through dissecting a particular resident cell type or signaling pathway involved in disease development and progression. Entering the era of systems biology, the new technologies that are constantly emerging allow us to dissect kidney diseases from a more refined and macroscopic perspective, such as the kidney local microenvironment (KLM).

The concept of a “microenvironment” has shaped our understanding of the pathogenesis of various diseases, especially tumorigenesis. Similarly, kidney lesions are not homogeneous across the kidney parenchyma amid disease progression. They often initiate at a specific site to form niche-like structures (2). Generally, a KLM is complex, heterotypic, and dynamic. It is comprised of diverse cellular and non-cellular components, such as injured tubular cells, activated fibroblasts, pericytes, inflammatory cells, endothelial cells, podocytes, and other cell debris, extracellular matrix, and circulated secreted factors (3). Although multi-omics approaches start to characterize the landscape of KLM at various dimensions (4, 5), the precise role of KLM in dictating kidney disease prognosis has largely been undetermined. In this regard, the Research Topic issue of “*Tissue microenvironment in kidney diseases*” is highly topical, aiming to highlight novel insights on the composition, structure, function, and regulation of KLM and seek innovative therapeutic strategies to treat kidney diseases.

Due to lifestyle changes, the spectrum of kidney diseases has been significantly altered. Metabolic diseases surpassed glomerular nephritis to become the leading cause of CKD. Compared with primary nephritis, these metabolic diseases no doubt run an additional detrimental external environment to accelerate kidney damage. For example, under diabetic stress, disturbance of metabolites in circulation directly or indirectly controls behavior changes of kidney resident cells such as death, survival, and autophagy. Podocytes are resident kidney cells and are the primary target cell type of diabetic kidney disease. In this issue, Sheng et al. illustrated that diabetes induced podocyte apoptosis and repressed autophagy in the chronically diseased glomeruli. After diabetic mice were treated with kaempferol, podocytes upregulated autophagic proteins LC3II, Beclin-1, and Autophagy-related 5&7, downregulated p62, and podocyte apoptosis was reduced. These protective effects are associated with the activity of the AMP-activated protein kinase/mammalian target of rapamycin (AMPK/mTOR) signaling pathways. As a result, the glomeruli microenvironment was improved by reducing albuminuria levels, alleviating mesangial matrix expansion, and amending glomerular basement membrane thickening. Besides podocytes, Yu, Wang et al. reported that hypoxia-inducible factor-1α (HIF-1α) protected against high glucose (HG)-induced tubular cell injury by promoting Parkin RBR E3 ubiquitin-protein ligase and PTEN-induced kinase 1 (Parkin/PINK1)-mediated mitophagy. Tubular cells are not a victim of CKD but are active participants. Under HG conditions, HIF-1α was upregulated in human proximal tubular cells. Inhibiting HIF-1α exacerbated hypoxia-induced mitochondrial dysfunction. Conversely, the HIF-1α-mediated protective effects were enhanced by scavenger N-acetylcysteine, a type of reactive oxygen species (ROS). HIF-1α-Parkin/PINK1-mediated mitophagy prevented tubular cell apoptosis and ROS production. These papers show how diseased podocytes or tubular cells contribute to KLM formation after CKD.

Diabetes could also cause kidney diseases progression by inducing cell senescence. As a critical cellular component of KLM, what active roles do senescent cells have in a chronically diseased kidney? Wang et al. found that senescent kidney tubular cells activated fibroblasts by secreting a growth factor called sonic hedgehog (Shh) to accelerate diabetic kidney disease (DKD) progression. They demonstrated that HG stimulated kidney tubular cells to secrete Shh. Meanwhile, the activity of SA-β-Gal in the cytoplasm of kidney tubular cells and the expression of senescence-related gene cell cycle regulators p16^INK4A^ and p21 were increased. D-Gal treatment in tubular cells increased the protein levels of Shh and p21 after DKD. Impressively, inhibition of Shh repressed fibroblast activation and tubular cell senescence. Cell senescence is a new topic in the field of kidney diseases, it certainly needs more robust evidence to evaluate how senescent cells impact KLM formation.

Other lifestyle factors also contribute to kidney disease. Yu, Mo et al. reported that high-fat diet induces chronic kidney damages *via* stimulating Wnt/β-catenin signaling. The role of Wnts in forming KLM is clear. Wnts are also related to cell senescence in the diseased kidney. Of note, the Wnt signaling pathway plays dual roles in acute and chronic stages of kidney disease, thus clinical applications targeting Wnts need to be carefully balanced.

Besides circulation factors, remote organ-organ communications also influence KLM formation, such as gut–kidney crosstalk. The CKD development is complex and heterogeneous. CKD patients showed differences in human host markers and gut microbiome signature compared to healthy individuals. Wehedy et al. reported the connections between human microbiome and CKD in this issue. From the perspective of dysbiosis, they described the changes in microbial diversity in CKD patients. The relationship between CKD and dysbiosis is bidirectional. Gut-derived metabolites and toxins affected CKD progression, while the uremic milieu affected the microbiota. Accumulated microbial metabolites and toxins resulted in kidney function loss and mortality risk increasing. Some renoprotective metabolites, such as short-chain fatty acids and bile acids, restored kidney functions and increased the survival rate of CKD patients. Specific dietary interventions to alter the gut microbiome could improve clinical outcomes of CKD. Low-protein and high-fiber diets increased the abundance of bacteria that produce short-chain fatty acids and anti-inflammatory bacteria. Ongoing human microbiome clinical trials may shed light on retarding CKD. Nevertheless, whether microbiota could directly penetrate the kidney membrane to exert their capacities in forming KLM needs further investigation.

Furthermore, in this issue, Deng et al. discussed the communication between resident kidney cells and infiltrated immune cells after acute kidney injury (AKI). After various acute insults, more immune cells are recruited and release chemo-cytokines to promote programmed cell death and phenotypic change of the resident cells. Meanwhile, the resident cells also promoted immune cell polarization. In the microenvironment after AKI, the bidirectional interaction between immune cells and resident cells determines the kidney fate. This is a typical cell-to-cell interactions in KLM, but completing the puzzle of AKI- or CKD-KLM may be a long journey. More studies focus on cell-cell, cell-matrix, and cell-secrete factors communications are needed to fully dissect KLM after various kidney diseases.

In summary, tissue microenvironment is an emerging concept and a new perspective in studying kidney diseases. A formed KLM reflects cellular–non-cellular factor communications in a diseased kidney and is also determined by various external factors in circulation and distant organs. Comprehensive and holistic consideration of intrinsic and extrinsic factors in KLM will be critical for constructing a warning system to monitor kidney disease progression. Supported by advanced technologies, we believe dissecting kidney diseases from the perspective of KLM will elevate our understanding of the pathogenesis to a new level.

## Author contributions

YG and DZ wrote the manuscript. SL and HF revised the manuscript. All authors read and approved the submitted version.

## References

[1] Foreman KJ Marquez N Dolgert A Fukutaki K Fullman N McGaughey M. Forecasting life expectancy, years of life lost, and all-cause and cause-specific mortality for 250 causes of death: reference and alternative scenarios for 2016-40 for 195 countries and territories. Lancet. (2018) 392:2052–90. 10.1016/S0140-6736(18)31694-5 30340847 PMC6227505

[2] Li L Fu H Liu Y. The fibrogenic niche in kidney fibrosis: components and mechanisms. Nat Rev Nephrol. (2022) 18:545–57. 10.1038/s41581-022-00590-z 35788561

[3] Fu H Gui Y Liu S Wang Y Bastacky SI Qiao Y. The hepatocyte growth factor/c-met pathway is a key determinant of the fibrotic kidney local microenvironment. iScience. (2021) 24:103112. 10.1016/j.isci.2021.103112 34622165 PMC8479790

[4] Dixon EE Wu H Sulvaran-Guel E Guo J Humphreys BD. Spatially resolved transcriptomics and the kidney: many opportunities. Kidney Int. (2022) 102:482–91. 10.1016/j.kint.2022.06.011 35788360

[5] Kuppe C Ibrahim MM Kranz J Zhang X Ziegler S Perales-Paton J. Decoding myofibroblast origins in human kidney fibrosis. Nature. (2021) 589:281–6. 10.1038/s41586-020-2941-1 33176333 PMC7611626

